# Management of paediatric burns in low- and middle-income countries: assessing capacity using the World Health Organization Surgical Assessment Tool

**DOI:** 10.1093/inthealth/ihz068

**Published:** 2019-10-15

**Authors:** Imogen K Thomson, Katie R Iverson, Simeon H S Innocent, Neema Kaseje, Walter D Johnson

**Affiliations:** Sydney Medical School, University of Sydney, NSW, AU; Program in Global Surgery and Social Change, Harvard Medical School, Cambridge, MA, USA; University of California Davis Medical Center, Sacramento, CA, USA; Faculty of Life Sciences and Medicine, King’s College London, London, UK; Global Initiative for Children’s Surgery, CH; Tropical Institute of Community Health and Development, Kisumu, KE; Emergency and Essential Surgical Care Programme, World Health Organization, Geneva, CH

**Keywords:** burns, global health, global surgery, paediatrics

## Abstract

**Background:**

Burns are a leading cause of global disease burden, with children in low- and middle-income countries (LMICs) disproportionately affected. Effective management improves outcomes; however, the availability of necessary resources in LMICs remains unclear. We evaluated surgical centres in LMICs using the WHO Surgical Assessment Tool (SAT) to identify opportunities to optimize paediatric burn care.

**Methods:**

We reviewed WHO SAT database entries for 2010–2015. A total of 1121 facilities from 57 countries met the inclusion criteria: facilities with surgical capacity in LMICs operating on children. Human resources, equipment and infrastructure relevant to paediatric burn care were analysed by WHO Regional and World Bank Income Classifications and facility type.

**Results:**

Facilities had an average of 147 beds and performed 485 paediatric operations annually. Discrepancies existed between procedures performed and resource availability; 86% of facilities performed acute burn care, but only 37% could consistently provide intravenous fluids. Many, particularly tertiary, centres performed contracture release and skin grafting (41%) and amputation (50%).

**Conclusions:**

LMICs have limited resources to provide paediatric burn care but widely perform many interventions necessary to address the burden of burns. The SAT may not capture innovative and traditional approaches to burn care. There remains an opportunity to improve paediatric burn care globally.

## Introduction

Burns are a leading cause of global morbidity and mortality, with an estimated 11 million people per year suffering new burns severe enough to warrant medical attention.^1^ Given that only a minority of burn patients ever seek medical care, this is likely to be a gross underestimation of the actual incidence.^2^ The burden of disease associated with burns is disproportionately borne by low- and middle-income countries (LMICs). LMICs account for an estimated 90% of burn cases, the majority of which occur in Africa and South East Asia.^3^ This is of particular concern as these countries may be insufficiently equipped to provide burn care, with close to 5 billion people currently lacking access to safe, effective and affordable surgical care in LMICs.^4^ Given that burns require rapid evaluation and treatment in order to obviate adverse sequelae, such as infection and contractures, this access gap has significant implications for patient outcomes.^5^

Children are the demographic at highest risk of sustaining burns, a pattern that is particularly pronounced in LMICs, where up to 80% of burn patients are <10 y of age.^6^ Children <5 y of age are at particularly high risk, due to normal developmental behaviours such as increasing independence but unsteady locomotion.^7,8^ Additionally, paediatric burns are more likely to be fatal, even when sustained over a smaller total body surface area (TBSA) compared with burns in older patients.^9^ When children do survive burns they are more likely to be permanently disabled as a result. This is partly attributable to young children most frequently sustaining burns to the hands and upper limbs, with normal growth patterns exacerbating contracture formation.^10^

Additional risk factors for paediatric burns help to explain their high incidence in LMICs. Rural communities are disproportionately affected and the incidence of burns among children correlates strongly with socio-economic factors such as poverty, household overcrowding and illiteracy.^2,11^ Most burns in LMICs occur in the home, where open flames are frequently used for cooking and warmth, explaining the annual peak in burns in some African regions during the cold Harmattan season from November to February.^12^ Traditional loose clothing worn by children in some communities may pose an additional risk in close proximity to open flames. As a result, children in LMICs are particularly vulnerable to burn-related injuries.

**Table 1:** Infrastructure and resources for paediatric burn care

	Income classification						Regional classification															Overall			
	LIC			MIC			AFRO			AMRO			EMRO			SEARO			WPRO						
Health centre level^a^	1	2	3	1	2	3	1	2	3	1	2	3	1	2	3	1	2	3	1	2	3	1	2	3	All
Facilities, n	35	147	137	73	469	260	40	186	249	2	27	35	3	14	21	48	330	73	15	59	19	108	616	397	1121
Infrastructure% of facilities																									
Electricity	14	48	78	64	75	82	15	59	77	100	78	89	67	43	90	63	72	86	73	78	90	48	68	81	71
Water	18	62	78	68	85	87	21	65	77	100	82	94	67	62	95	73	91	97	53	64	95	52	80	84	79
Oxygen	33	63	81	67	83	87	32	69	81	100	81	94	67	64	85	71	84	94	60	76	84	56	78	85	78
Sterilizer	50	73	75	62	71	86	51	75	82	100	58	84	100	86	65	69	72	86	33	59	84	55	71	82	74
Equipment and supplies % of facilities																									
Sterile gloves	58	69	77	86	73	91	58	72	90	100	67	77	100	100	57	89	70	85	79	76	95	77	72	87	76
Nasogastric tube	42	58	65	52	59	80	42	60	76	100	65	74	100	93	52	46	58	79	60	52	74	49	59	75	64
Essential i.v. fluids	26	43	48	10	27	54	20	41	51	100	41	57	67	79	38	6	23	58	7	25	58	15	31	52	37
Resuscitation bag/mask	20	45	61	23	40	55	20	43	52	50	46	68	67	71	45	18	40	73	27	38	58	22	42	57	45
Essential suturing	32	48	57	59	55	75	35	532	72	100	44	57	100	71	48	63	54	74	40	44	58	51	53	69	58
Essential surgical	49	58	64	32	53	84	45	62	80	50	56	63	67	86	48	33	51	84	20	44	74	37	54	77	61
Essential intubation	6	24	34	4	16	40	3	27	32	100	11	40	67	21	48	0	12	51	0	24	53	5	18	38	24

Essential equipment and supplies for suturing: needles, needle holder, scissors and sutures. Essential surgical equipment and supplies: scalpel blades, retractor and artery forceps. Essential supplies for provision of i.v. fluids: infusion sets, cannulas and infusion bags. Essential supplies for paediatric intubation: paediatric laryngoscope, paediatric endotracheal tube and paediatric Magill forceps. Oxygen: oxygen concentrator or oxygen cylinder.

^a^Health centre classification: 1, community health centre or other primary care facility; 2, district, provincial or regional hospital; 3, general, non-governmental organization, private or mission hospital.

Appropriate management has been demonstrated to be highly effective in reducing the morbidity and mortality associated with paediatric burns. In high-income countries (HICs), a combination of resuscitation, acute care and long-term wound management has meant that children with significant burns of up to 60% TBSA frequently survive, with morbidity reduced by rehabilitation.^13,14^ In contrast, in resource-poor countries, burns involving as little as 20% TBSA are often fatal, particularly if affecting the airway, and few children with burns >45% TBSA survive.^10,15^ Common causes of death include delay in wound management and closure, infection, inadequate fluid resuscitation and insufficient nutritional support, many of which can be prevented with effective and timely burn care.^10^

The Surgical Assessment Tool (SAT; also known as the Tool for Situational Analysis to Assess Emergency and Essential Surgical Care) was designed by the WHO Emergency and Essential Surgical Care Programme and partners to assess gaps in the availability of surgical care in resource-constrained health facilities.^16^ The WHO’s commitment towards the Sustainable Development Goals (SDGs) encompasses surgery and a reduction in paediatric mortality as critical components of SDGs 3.2 and 3.8: reducing preventable child mortality and achieving universal health coverage. Given that burns account for a considerable proportion of paediatric hospital admissions in LMICs, and typically require surgical care, timely and effective burn management for children is a requisite element of the SDGs. However, these targets cannot be achieved without a detailed understanding of the capacity of facilities in LMICs to provide paediatric burn care. In order to identify opportunities to optimize patient management, we sought to use the WHO SAT database to assess the capacity of facilities in LMICs to manage paediatric burns.

## Methods

We performed a retrospective review of the WHO SAT database, a collaborative initiative led by the WHO Emergency and Essential Care (EESC) Programme. The WHO SAT is completed voluntarily by clinicians working worldwide, with responses sought through WHO country offices (which then contact facilities in their country) and from members of the WHO Global Initiative for Emergency and Essential Surgical Care network, both through electronic and paper submissions. Responses are compiled into a database by the WHO EESC team. The SAT comprises 108 questions across four domains: infrastructure, human resources, interventions and equipment and supplies.^16^ The SAT has evolved across three iterations, with the first two having no significant differences for the purpose of this study. The third version (SAT3) includes additional detailed questions, which were not considered for the purpose of this analysis.

The SAT (all versions) was completed 1489 times between October 2010 and April 2015 by clinicians from facilities in 63 countries, comprising the SAT database. Inclusion criteria for this study were facilities in LMICs with some surgical capacity (as indicated by the presence of at least one major or minor operating room) and providing paediatric surgery (as defined by those facilities performing at least one paediatric operation per year).

Countries were grouped and analysed according to World Bank income classifications of gross national income per capita per year (low income [<US$995] and middle income [including both lower-middle and upper-middle income; US$996–12 055])^17^ and according to the WHO regional classification: Africa (AFRO), the Americas (AMRO), the Eastern Mediterranean (EMRO), Europe (EURO), South-East Asia (SEARO) and the Western Pacific (WPRO).^18^

Facilities were grouped and analysed based on facility type: primary (health centres or clinics), secondary (regional, district or provincial hospitals) and tertiary (general, teaching, private and non-governmental organization hospitals). Duplicate entries were excluded.

Only variables relevant to the provision of paediatric burn care were included. Although not directly utilized in acute burn care, suturing supplies were included as a useful comparison for the availability of resources required for burn care as well as for the necessity of suturing equipment in long-term surgical management of burns. Equipment and supplies were grouped according to purpose: suturing (needles, needle holder, scissors, sutures), surgery (scalpel blades, retractor, artery forceps), provision of intravenous (IV) fluids (cannulas, infusion set, infusion bag) and paediatric intubation (paediatric laryngoscope, paediatric endotracheal tube, paediatric Magill forceps). We considered equipment and supplies to be present only if clinicians indicated that they were consistently available for all patients, and did not consider equipment, supplies and resources that were inconsistently available. Some specific resources necessary for burn care, such as dressings and silver sulfadiazine cream, were not recorded in the SAT and thus could not be included in this study.

Each variable was summarized by income classification, income and region and expressed as the mean of the facilities responding (human resources, facility factors) or the percentage of facilities indicating that the variable was present (equipment, supplies, infrastructure) or performed (interventions). Distance travelled was used to indicate barriers to accessing care and was calculated for interventions performed by <75% of facilities overall. This was measured as the proportion of clinician respondents who indicated that an average patient travelled >80 km to access care at their facility. Statistical analyses were conducted in Stata 14.2 (StataCorp, College Station, TX, USA).

## Results

**Figure 1:**
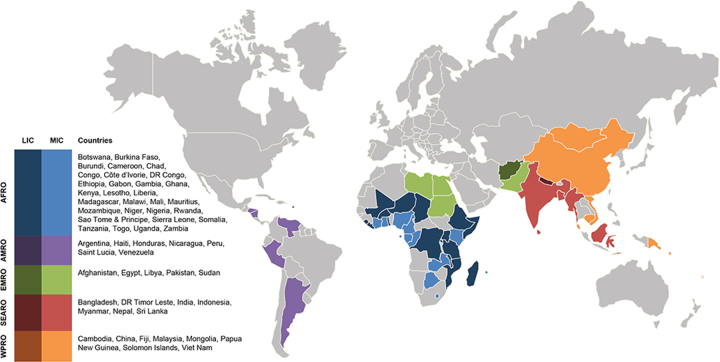
Distribution and income classification of responding countries.

A total of 1121 facilities from 57 countries and five WHO regions (AFRO, AMRO, EMRO, SEARO, WPRO) met the inclusion criteria, with a mean of 20 facilities per country. Table 1 highlights that there were significantly more facilities per region responding from AFRO (475) and SEARO (451). Similarly, the majority of facilities were in middle-income countries (802, including 775 low-middle income and 27 upper-middle income), with 319 respondents from low-income countries. On average, facilities had 147 beds and performed 485 paediatric operations per year. Figure 1 shows the distribution of facilities and countries across regions and income categories.

Table 1 highlights the relationship between resources and infrastructure, and region and income category. The likelihood of reliable access to key infrastructure and equipment generally increased across income categories and was highest in tertiary-level centres, with primary-level health centres with notably fewer resources. LICs had the fewest resources across all domains, particularly with respect to consistent access to critical utilities such as running water (57%) and electricity (64%). Considering the equipment and supplies necessary for surgical and burn care, sterile gloves (78%) and essential equipment for surgery (61%) were the most widely available. Supplies for the management of children’s airways, such as paediatric intubation equipment (24%) and masks and bags for resuscitation (45%), were least available, along with essential equipment for the provision of IV fluids (37%). The availability of key equipment and resources was variable, but generally similar between regions.

Human resources varied by income, hospital level and region (Table 2). Overall, non-specialist physician surgeons were nearly as common as specialist surgeons (5.0 vs 5.1 per facility), while non-physician (nurse or other health professional) anaesthesiologists were far more common than specialist and physician non-specialist anaesthesiologists (4.1 vs 1.6 vs 1.3 per facility). LICs and secondary-level facilities particularly utilized non-specialist surgeons and anaesthesiologists. However, even in tertiary-level centres in MICs, non-physician anaesthesiologists predominated (6.4 per facility) over specialist physician (4.6) and non-specialist physician (3.8) anaesthesiologists.

**Table 2:** Human resources for paediatric burn care

	Income classification				Regional classification										Overall		
	LIC		MIC		AFRO		AMRO		EMRO		SEARO		WPRO				
Health centre level^a^	2	3	2	3	2	3	2	3	2	3	2	3	2	3	2	3	All
Facilities, n	147	137	469	260	186	249	27	35	14	21	330	73	59	19	616	397	1013
Human resources, mean no. per facility																	
Surgery																	
Specialist physician surgeons	1.5	5.0	2.1	13.9	2.0	6.7	1.1	10.2	0.9	41.7	1.1	11.9	6.3	8.5	1.9	10.3	5.1
Non-specialist physician surgeons	2.1	3.2	3.1	11.2	3.5	5.0	0.1	0.9	3.5	29.1	2.0	17.9	5.1	6.1	2.8	8.5	5.0
Other health professional providing surgical care	2.2	3.8	0.9	4.3	2.2	3.3	0.2	1.3	0.9	16.5	0.6	4.8	2.1	2.0	1.3	4.1	2.3
Anaesthesia																	
Specialist physician anaesthesiologist	0.6	1.2	0.7	4.6	0.5	1.5	1.0	2.9	0.4	18.9	0.4	4.0	1.8	3.5	0.6	3.3	1.6
Non-specialist physician anaesthesiologist	0.2	0.7	0.6	3.8	0.4	1.6	0.2	0.3	0.3	7.2	0.4	5.6	1.0	2.8	0.5	2.7	1.3
Other health professional providing anaesthesia care	2.3	4.3	3.5	6.4	2.9	4.7	1.3	10.4	1.5	10.6	3.9	5.5	2.8	2.4	3.2	5.6	4.1

^a^Health centre classification: 2, district, provincial or regional hospital; 3, general, non-governmental organization, private or mission hospital.

Of the general burns-related interventions considered, wound debridement (90%) and acute burn care (86%) were the most widely performed overall (Table 3). Specific initial first aid measures for burns—acute burn care and resuscitation—were performed in 86% and 81% of facilities, respectively. Procedures were generally widely available in secondary and tertiary centres and were more available in LICs than MICs. Procedures that may be required for long-term management of burns were performed less frequently, with amputation performed by 42% of secondary and 72% of tertiary facilities and contracture release and skin grafting performed by 31% of secondary and 63% of tertiary facilities. A large minority of patients travelled long distances to access care, with 32% typically travelling >80 km to access facilities providing amputation (36% in LICs, 28% in MICs) and 31% typically travelling >80 km to access facilities providing skin grafting and contracture release (41% in LICs, 26% in MICs).

**Table 3:** Interventions for burn care

	Income classification								Overall			
	LIC				MIC							
Health centre level^a^	1	2	3	All	1	2	3	All	1	2	3	All
Facilities, n	35	147	137	319	73	469	260	802	108	616	397	1121
Interventions, % of facilities												
Resuscitation with advanced life support	69	94	96	92	54	72	92	77	59	77	94	81
Acute burn care	83	96	92	93	65	81	90	83	71	85	90	86
Wound debridement	69	99	96	89	73	87	97	89	72	89	97	90
Any anaesthesia	54	98	98	93	24	48	96	61	32	60	97	70
General inhalational anaesthesia	44	73	82	74	21	34	67	44	28	43	72	52
Ketamine intravenous anaesthesia	51	97	98	92	22	47	94	60	32	59	95	69
Amputation	–	73	87	73	–	32	64	41	–	42	72	50
Contracture release and skin grafting	–	44	74	52	–	26	58	36	–	31	63	41

^a^Health centre classification: 1, community health centre or other primary care facility; 2, district, provincial or regional hospital; 3, general, non-governmental organization, private or mission hospital.

Anaesthesia was variably available, with 60% of secondary and 97% of tertiary facilities providing any anaesthesia. In both cases this was driven by the widespread availability of ketamine IV anaesthesia, provided by 59% of secondary and 95% of tertiary facilities, with general inhalational anaesthesia being less widely used. There were many disparities between interventions performed and access to equipment deemed necessary by the WHO to perform these procedures, such as resuscitation with advanced life support being performed by 81% of facilities, but resuscitation bags and masks being available in 45% of facilities and supplies for paediatric intubation available in only 24% overall. This pattern was reflected across most interventions and accompanying supplies (Tables 1 and 3).

## Discussion

Our results indicated that there were numerous strengths in the provision of paediatric burn care in LMICs, such as the widespread availability of important interventions such as wound debridement and acute burn care. However, there remains opportunity for improvement across all WHO regions and income categories. These findings are in keeping with, and expand upon, other research in this field. A review of paediatric burn injuries in sub-Saharan Africa highlighted barriers to care provision similar to our results.^10^ Inadequate airway management and insufficient fluid resuscitation were two key factors contributing to high mortality in the acute stage of paediatric burn care.

The International Society for Burn Injuries guidelines highlight the crucial role of airway management in the acute care of the thermally injured patient.^19^ Airway stabilization, particularly early intubation, is especially important when treating paediatric patients, whose anatomy places them at greater risk of obstruction. In LMICs, airway burns strongly correlate with poor outcomes, with mortality associated with smoke inhalation injuries being reported as high as 68.7%.^7^ Despite the importance of airway management, our study indicated that the equipment necessary for paediatric intubation was rarely available in hospitals in LMICs. Similarly, despite the importance of oxygen therapy in burn care, supplementary oxygen was inconsistently available among the facilities studied, with access particularly limited in LICs. A study of referrals to a tertiary burn centre in Durban, South Africa highlighted that initial burn management in LMICs is often inadequate, with a lack of resources playing a role.^20^

Fluid resuscitation is another critical aspect of first aid in burn management, particularly in the acute care of paediatric patients, who have the potential to rapidly deteriorate and develop burn shock.^21^ Our data show that many facilities lack the capacity to reliably provide simple IV fluids. Insufficient resources are compounded by other factors that may impact the provision of fluid support, with a recent review highlighting that even if the necessary routine equipment and supplies are available, there are numerous practical barriers to the timely administration of fluid resuscitation, such as unclear or inconsistent guidelines.^22^ The combination of these factors is likely to impact the overall quality of care provided to paediatric burn patients in LMICs and could potentially impact mortality rates. However, this study did not consider the use of enteral fluids for resuscitation, as this is not captured in the WHO SAT, although Rode et al.^22^ and Venter et al.^23^ have shown this to be a useful and effective alternative in some circumstances.

While appropriate first aid is key in reducing the initial mortality associated with paediatric burns, definitive management plays a similarly important role in reducing the long-term sequelae associated with these injuries. A recent study of the ReSurge International Database highlighted that children are disproportionately affected by contractures, with younger age at the time of the burn and longer delay in the time to corrective surgery correlating with poorer outcomes. Patients in LMICs typically wait 4–6 years for contracture release, with longer delays leading to a greater dysfunction, resulting in a 12.5 times higher likelihood of a poor outcome in LMICs compared with HICs.^24^ Our results indicate that 41% of secondary and tertiary facilities performed contracture release and skin grafting (31% of secondary centres and 63% of tertiary centres). While 41% of facilities may be appropriate for more complex interventions, respondents also indicated that the average patient travels >80 km to access care at 31% of facilities (41% in LICs, 26% in MICs). Distance is a notable impediment to care provision in countries with large or isolated rural populations, regardless of income status, but is particularly challenging for those that have suboptimal infrastructure or additional geographic barriers. This is important in the context of burn care, with burns disproportionately affecting rural populations and treatment often being time-critical. The combination of these factors may pose significant challenges to children seeking to access important aspects of long-term burn care, and additional research on the equitable accessibility of long-term interventions for patients with burns is needed.

One theme in our results was the discrepancy between the reported burn care interventions provided by facilities in LMICs and the availability of the equipment and supplies necessary to perform them. This was particularly relevant in the context of anaesthesia care. Our data show that surgical procedures were far more widely available than anaesthesia, which has significant implications for paediatric burn care. Burns are profoundly painful, particularly during necessary interventions such as debridement. This procedural pain has been associated with acute and long-term stress disorders in children, with burn care practice guidelines now emphasizing the importance of adequate analgesia and anaesthesia, especially during interventions that may cause additional pain and in children.^19,25^ Although we did not have access to data on the availability of analgesic medications, the relatively limited access to anaesthesia is a particular area that warrants concern and merits further attention.

Our results also identified ways in which care in LMICs differs from that typically provided in HICs but remains highly effective and could be further expanded upon to improve access for patients. In contrast with anaesthesia care in HICs, which is typically provided by specialist anaesthesiologists using general inhalational anaesthesia, our data emphasize the important role that generalist physician and paramedical workers play in anaesthesia care in LMICs.^26^ These clinicians are often highly skilled and work in conjunction with or independent of anaesthesiologists to significantly increase access to anaesthesia care in resource-limited settings. Similarly, data from Mozambique highlight that task-shifting of some procedural obstetric care to trained non-physician associate clinicians has led to much improved access to care, particularly in rural areas, without a clinically significant difference in patient outcomes.^27^ Greater use of trained, non-specialist physician or paramedical healthcare workers in anaesthesia and surgical care may represent one avenue to improve access to paediatric burn care in LMICs.

The use of ketamine i.v. anaesthesia is related and also key to the provision of anaesthesia care. A recent study by Burke et al.^26^ highlighted the safety and feasibility of ketamine anaesthesia administered by trained healthcare workers in Kenya when an anaesthesiologist is unavailable. It was noted that protocols providing for the administration of ketamine anaesthesia by non-anaesthesiologists may increase access to surgery and anaesthesia in geographically isolated LMICs, in keeping with the findings of our study, without compromising patient care. Droussi et al.^28^ also noted that ketamine may be safe and effective when used as an analgesic to facilitate dressing changes and wound care for children with burns in LMICs. These studies, and the data that our research demonstrates, highlight the importance of access to this medication.

Although our results demonstrate a lack of resources necessary to perform the procedures indicated in paediatric burn care in many facilities in LMICs, there are additional explanations for this finding that warrant consideration. First, one limitation of our study is that while the SAT considered the resources deemed necessary by the WHO, it may not have adequately captured alternatives being used in LMICs. These include both clinicians utilizing limited available equipment and supplies in innovative ways to care for patients, as well as the use of traditional medicine in burn management.^29^ There is evidence that select traditional practices, such as the application of topical papaya, may have some efficacy in paediatric burn management in resource-limited settings.^30^ Given that the SAT did not include space for clinicians to elaborate on exactly how paediatric burns are managed in practice in their facility, these details may have been overlooked. Additional data on other non-surgical treatments for burn care, such as burn dressings, were not captured by this study, and additional data about these important measures are also needed. Lastly, we also did not consider resources or infrastructure that were unreliably or inconsistently available..

There are other limitations of our study that warrant acknowledgment. First, the majority of children who suffer from burns in LMICs do not present to a hospital.^2,11^ As a result, additional research into the management of paediatric burns in the community setting is necessary in order to adequately capture all aspects of burn care. Although we classified hospitals based on primary, secondary and tertiary centres for the purposes of analyses, it is noted that there may be significant variations within these categories. Second, it is likely that facilities that had access to and completed the SAT are relatively well resourced. This is a conservative reporting bias that likely results in an overestimation of the provision of burn care. Paediatric burn care in LMICs. Third, data were not collected in a cross-sectional manner, so countries were not proportionately represented, and many LMICs were not represented at all. In the future, seeking more targeted responses may be necessary to correct this. Finally, data were collected during a 4.5-y period, commencing in October 2010. As a result, while the overarching findings remain extremely pertinent, regional variations in results may reflect broader external factors, such as international aid and investment in certain regions during this time, which may have subsequently shifted by the time of publication of this study. We also did not consider strategies for prevention of paediatric burns, which must be recognized as the most important aspect of burn care.

## Conclusions

Paediatric burns are a significant cause of global morbidity and mortality and disproportionately affect children in LMICs. Although burn injuries are frequently associated with poor short- and long-term consequences within this population, many of these outcomes may be improved with timely and appropriate management. Our research highlighted particular areas warranting additional attention, including paediatric intubation and anaesthesia care, fluid resuscitation and ensuring that procedures necessary for long-term care, such as contracture release and skin grafting, are accessible for patients. The WHO SAT is a useful tool for evaluating the capacity of facilities in LMICs to care for paediatric burn patients, but there are important additional factors in care provision that this survey did not capture which warrant further evaluation. The forthcoming WHO–Global Initiative for Children’s Surgery Paediatric Surgical Assessment Tool may be helpful in this setting.
